# Improved visualization and quantification of 4D flow data using divergence-free wavelets

**DOI:** 10.1186/1532-429X-15-S1-E26

**Published:** 2013-01-30

**Authors:** Frank Ong, Martin Uecker, Umar Tariq, Albert Hsiao, Shreyas S Vasanawala, Michael Lustig

**Affiliations:** 1 Department of Electrical Engineering and Computer Science, University of California, Berkeley, Berkeley, CA, USA; 2 Department of Radiology, Stanford University, Stanford, CA, USA

## Background

4D flow MRI has the potential to provide global quantification of cardiac flow in a single acquisition [Hsiao, 2012]. However, 4D flow data are often compromised by low velocity-to-noise ratio, potentially caused by MRI acceleration or unfitting vencs. Since blood flow is approximately divergence-free, noise level can be reduced by removing divergence from noisy flow data [Song, 1993] [Busch, 2012]. On the other hand, strict enforcement of divergence-free condition distorts flow around edges as discrete approximation of flow near edges creates divergence. In this current work, we aim to provide an adjustable and fast operation of imposing multi-scale divergence-free conditions on flow data by using divergence-free wavelets. In addition, we utilize the sparsity of flow data in divergence-free wavelet domain [Deriaz, 2006] for further denoising by performing wavelet shrinkage [Donoho, 1995].

## Methods

Divergence-free wavelets were used to transform flow data into diverence-free and non-divergence-free components in wavelet domain. A threshold was applied on non-divergence-free components to reduce divergence except for high magnitude divergence such as those near edges. A lower threshold was also applied on divergence-free components to sparsify the coefficients. To further validate the improvement,* in vivo* 4D cardiac flow data were acquired in 8 patients with 20 heart phases, 122-144 slices and an average spatial resolution of 1.56 mm,1.56 mm,1.43 mm on a 1.5T GE Scanner. Flow data were extracted from eddy-current corrected phase of reconstructed images using L1-SPIRiT [Lustig, 2010]. Segmentations were done manually on aorta and pulmonary trunk. Net flow rate (volume/time) and regurgitant fraction (%) were then calculated for each segmentation. Flow inconsistency was defined as the absolute difference between flow rates in the aorta and pulmonary trunk and should equal zero for noiseless data.

## Results

**Figure 1:**
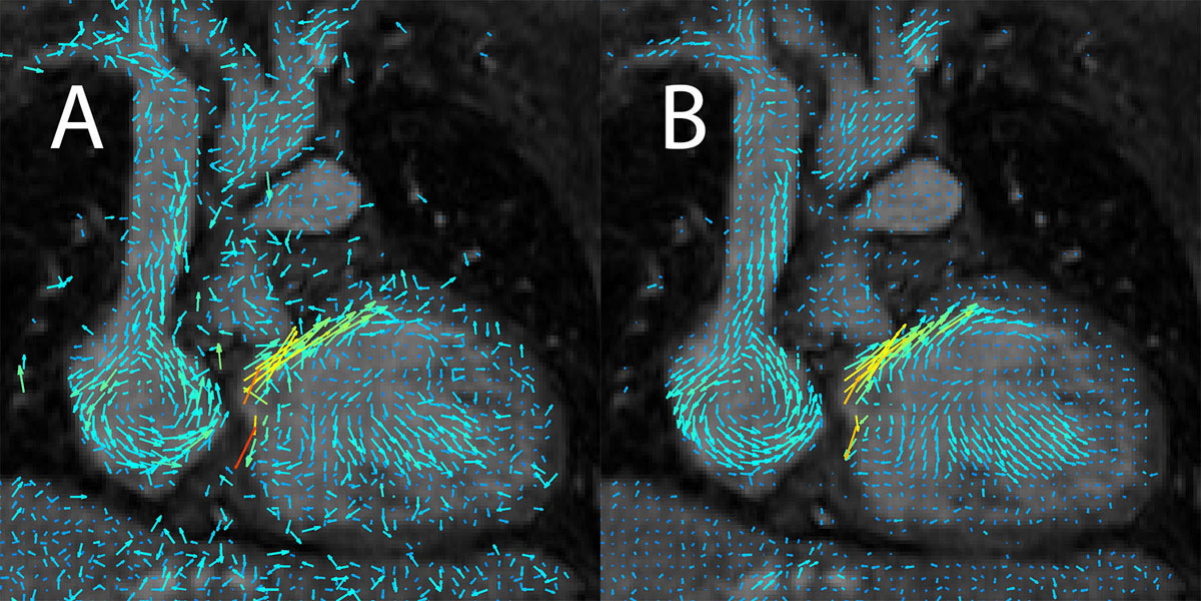
Visualization of coronal cross-section of cardiac flow featuring the superior vena cava, before denoising (A) and after denoising (B). All images are masked by the magnitude image.

**Figure 2:**
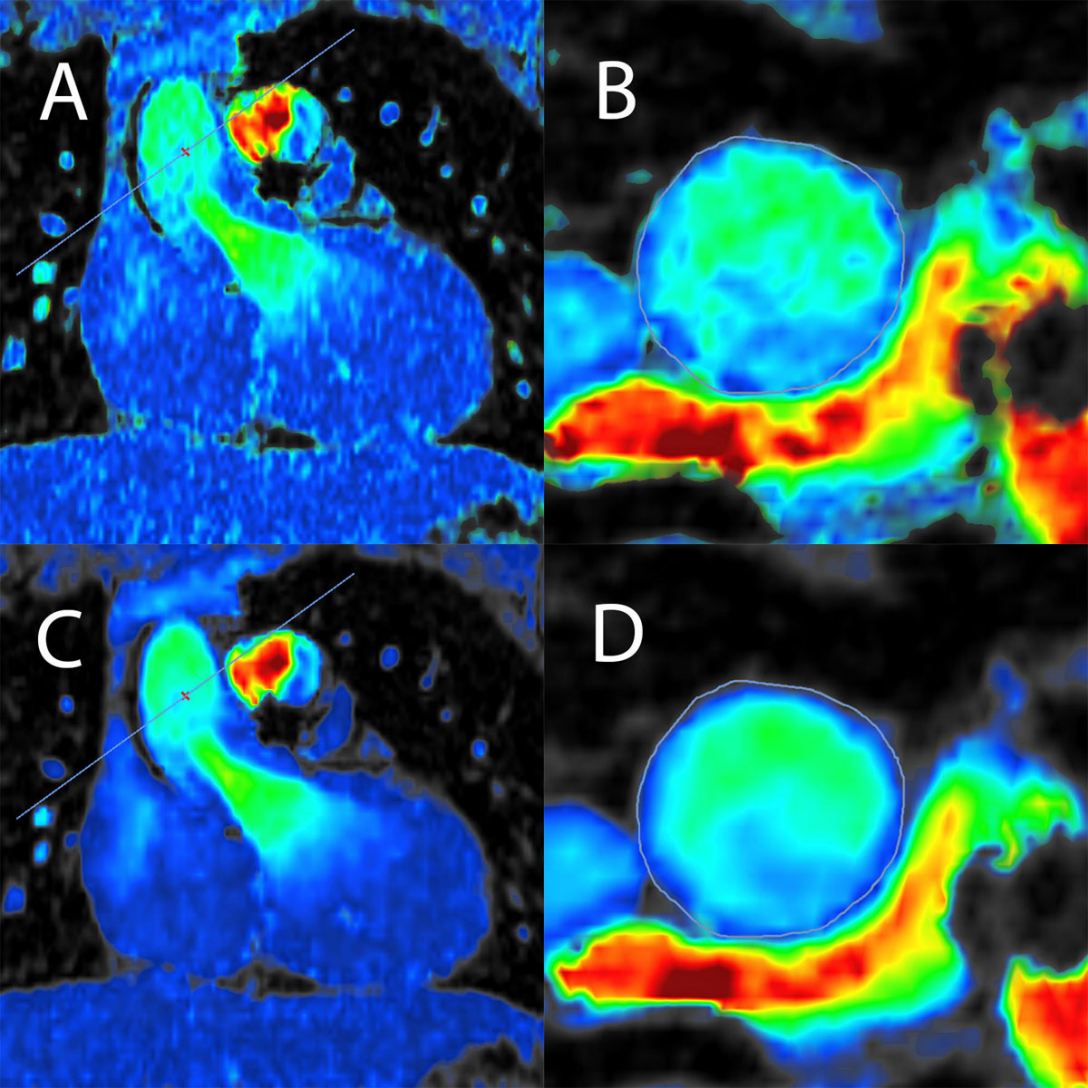
Visualization of cardiac flow magnitudes before denoising (A), with closeup of segmented aorta slice (B) and after denoising (C), with closeup of segmented aorta slice (D). All images are masked by the magnitude image.

Both figure 1 and 2 show improved visualization after post-processing. Studies were evenly separated into a group with regurgitant fractions less than 5% (mean net flow = 2.945 l/min) and a group with regurgitant fractions more than 30% (mean net flow = 2.212 l/min). For the first group, the average flow inconsistency before denoising was 0.395 l/min and after denoising was 0.353 l/min, yielding a 10.7% improvement. Average change in regurgitant fraction was 0.08%. For the second group, the average flow inconsistency before denoising was 1.151 l/min and after denoising was 0.926 l/min, yielding a 19.5% improvement. Average change in regurgitant fraction was 1.88%. Each processing of a 3D volume ran within half a minute in Matlab on a 2.8 GHz Core2Duo laptop with 4GB of RAM.

## Conclusions

Divergence-free wavelet denoising was shown to enhance the visual quality of flow data while preserving quantification of flow at aorta and pulmonary trunk. The processing was also shown to be fast and adjustable for enforcing divergence-free constraints.

## Funding

SRC, NIH grants P41RR09784 and American Heart Association 12BGIA9660006.

